# An Assessment of the Efficacy of Commercial Air Ionizer Systems Against a SARS-CoV-2 Surrogate

**DOI:** 10.3390/microorganisms13030593

**Published:** 2025-03-04

**Authors:** Nachiket Vaze, Brittany Gold, Douglas Lindsey, Matthew D. Moore, Petros Koutrakis, Philip Demokritou

**Affiliations:** 1 Nanoscience and Advanced Materials Center, Environmental and Occupational Health Sciences Institute, Rutgers University, Piscataway, NJ 08854, USA; nv282@rutgers.edu; 2 Department of Food Science, University of Massachusetts Amherst, Amherst, MA 01003, USA; blgold@umass.edu (B.G.); mdmoore@umass.edu (M.D.M.); 3 The Electric Power Research Institute, Palo Alto, CA 94304, USA; dlindsey@epri.com; 4 Department of Environmental Health, Harvard T.H. Chan School of Public Health, Boston, MA 02215, USA; petros@hsph.harvard.edu

**Keywords:** air ionizers, SARS-CoV-2, antiviral, bioaerosols, disinfection, MS2

## Abstract

Airborne transmission has been implicated as a major route for the spread of microorganisms, causing infectious disease outbreaks worldwide. This has been emphasized by the recent COVID-19 pandemic, caused by the SARS-CoV-2 virus. There is thus an unmet need to develop technologies that arrest the spread of airborne infectious diseases by inactivating viruses in the air. In this study, the efficacy of two commercially available air ionizer systems for inactivating the bacteriophage MS2, which has been utilized as a surrogate of SARS-CoV-2 as well as a surrogate of noroviruses, was assessed. An experimental test apparatus similar to an HVAC duct system was utilized for the efficacy testing. Each of the two ionizer devices was challenged with viral aerosols of the bacteriophage MS2. The results indicate that the two ionizers were able to reduce the concentration of bacteriophage MS2 virus in the air by 82.02% and 81.72%, respectively. These results point to the efficacy of these ionizer devices in inactivating airborne microorganisms and thus making them an important tool in arresting the spread of infectious diseases. More studies are needed to assess their efficacy against other important airborne viruses such as influenza and strains of the SARS-CoV-2 virus.

## 1. Introduction

The COVID-19 pandemic has highlighted the need to develop new technologies for arresting the spread of infectious diseases [1,2,3,4]. COVID-19 is caused by the SARS-CoV-2, a coronavirus of the betacoronavirus family [5,6]. Although preventative measures such as masks and social distancing may help in reducing the overall rate of spread of the SARS-CoV-2 virus, there is still a need for effective intervention methods that can kill the virus in the air. The airborne spread of the virus is primarily through bioaerosols. Bioaerosols are suspensions of airborne particulate matter of biological origin, which includes microorganisms (such as viruses and bacteria) and the products of these organisms [7]. To this effect, technologies such as ultraviolet light, other light-based devices, electrical plasma discharges, ozone, air ionizers, and nanotechnology-based antimicrobial platforms, such as Engineered Water Nanostructures (EWNS) are emerging as contenders in this space [8,9,10,11,12,13,14,15,16]. These technologies have their own advantages and disadvantages, and their efficacy in inactivating airborne viruses, especially in air, needs to be investigated further.

One such technology, widely used commercially, is air ionizers [17]. These ionizers utilize electrical energy to produce positively and negatively charged ions. The ions generated are known to damage cell membranes by physical and chemical processes [18]. In addition to the ions, they may also produce ozone, which is a known microbicidal agent, and other reactive oxygen species (ROS). However, many of the ionizers in the market today claim to pass UL2998, which limits ozone to no more than 5 ppb (parts per billion) [19,20].

Lee et al. (2014) demonstrated the efficacy of a duct-installed air ionizer system, reporting an 85% reduction in the concentration of airborne *Staphylococcus epidermidis* [21]. Ionizers have also been utilized in conjunction with HEPA (High Efficiency Particle Arresting) filters, in order to electrically charge bioaerosol to increase the air filtration efficiency [17]. Fletcher et al. (2007) suggested that the bactericidal effect of the air ions is primarily due to the electroporation of bacteria [22].

The literature regarding the antiviral effects of ionizers is sparse. A study of the efficacy of ionizers against the Porcine Reproductive and Respiratory Syndrome (PRRS) virus indicated up to a 96% reduction in viral aerosol concentration [23]. In a lab-based study of viral aerosols produced inside a small enclosed chamber, ten minutes of air ionization at ion concentration of 10^6^ ions/cm^3^ led to 4-log reduction in the concentration of airborne mammalian reovirus [24].

A recent review of the methods to reduce the probability of the airborne spread of COVID-19 in mechanically ventilated systems and enclosed spaces has emphasized the fact that most of the studies that assess the efficacy of ionization-based systems rely on experiments with smoke particles, or other solid particles, instead of actual viral particles in air [25]. Hence, there is a knowledge gap in terms of the ability of ionizers to inactivate airborne viruses, including SARS-CoV-2.

The current study assesses the efficacy of two commercially available air ionizer systems that are meant to be installed inside existing heating, ventilation, and air conditioning (HVAC) ducts. The ionizers were challenged with the aerosols of the bacteriophage MS2 virus, a non-enveloped virus widely utilized for antiviral testing as a surrogate of SARS-CoV-2 [26,27,28,29]. Bioaerosols were exposed to the ions generated by the two ionizers in a custom-built duct-testing apparatus, and samples of air inside the duct downstream of the ionizers were taken and assessed for viral activity with and without the ionizers turned on. This study design will help with understanding the efficacy of these ionizers in a potential real-world setting.

## 2. Materials and Methods

### 2.1. Ionizers

This study assessed two commercially available ionizer devices for their efficacy in inactivating airborne MS2:

The first ionizer device (termed as device A) is a two-tube commercial quality unit intended for installation in HVAC duct systems for residential and commercial applications. It consists of a square front panel with mounting screws for installing the device inside a duct, perpendicular to the direction of the airflow. The device is operated at 120V AC. It draws up to 16 watts of power. The device has five levels of ion generation. For the purposes of this study, the ionizer was operated at the highest level (5). The airflow capacity was rated as up to 4000 cubic feet per minute (cfm).

The second ionizer device (termed as device B) tested is a duct-mounted needlepoint bipolar ionization system. The device has a cylindrical cross section, with the front panel consisting of screws for installing the device inside a duct, perpendicular to the airflow. The rated wattage for this device is 4 watts. The maximum ion generation output is 4 × 10^8^ ions/cc. The airflow capacity of the device is 0–4800 cfm.

### 2.2. Experimental Setup for Ionizer Testing

**Figure 1:**
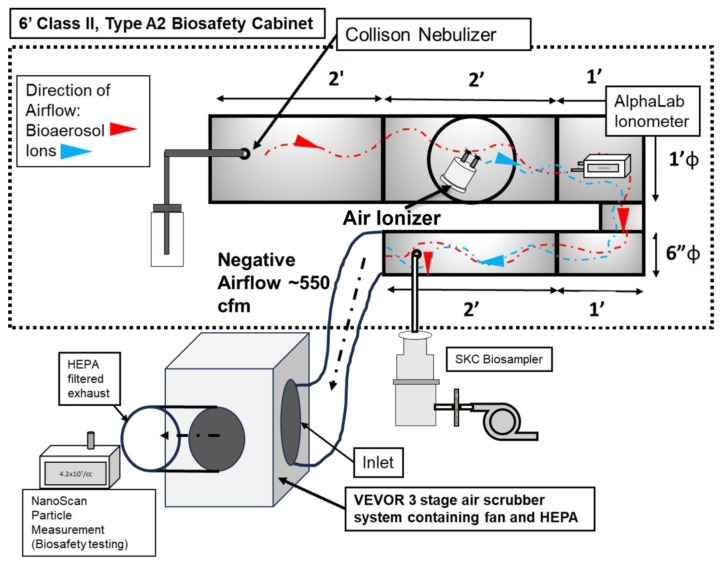
Schematic of the experimental setup, showing the various components for ion measurement and antiviral efficacy testing.

Figure 1 illustrates the experimental setup developed for the ionizer testing. The operational airflow was provided by a commercially available 3-stage High Efficiency Particle Arresting (HEPA) filtration unit with a built-in fan (Model No: ZHP2301-550, VEVOR, Rancho Cucamonga, CA, USA). The VEVOR unit is an air particle and gas scrubber that features a three-stage filtration system consisting of a pre-filter (MERV-10), a carbon filter, and an H13 HEPA filter [30]. This unit provided the necessary airflow for the experiment as well as HEPA filtration. The fan provides up to 550 cfm airflow. The fan/filtration unit was connected to the stainless-steel duct system (placed inside a biological safety cabinet (BSC).

The duct system was custom designed and fabricated with steel components (Figure 1). There were certain criteria and limitations associated with the design. The chamber had to be entirely inside a 6-foot, Class II, Type A2 biosafety cabinet (BSC), since there would be aerosols of BSL-2-level microorganisms involved. The ionizers were installed in the duct as recommended by the manufacturers. Total duct length was to also be maximized so as to increase the contact time of ions with the viral aerosol. Thus, the setup consists of the following components: (1) The aerosol injection section: a 12-inch-diameter, 2-foot-long duct-shaped section consisting of a single piece of steel. Six inches from its edge, centrally, it contained a port for the injection of bioaerosol. This port was connected to the output of the Collison nebulizer by screwing in the outlet of the Collison nebulizer. (2) The air ionizer section: This is a custom fabricated, 12-inch-diameter, 2-foot-long duct-shaped section made from steel. It has a window for inserting the test device into the duct. The test ionizer is inserted into the window and screwed into place. A gasket around the edge of the device provides a seal. (3) The ‘U’-shaped connector section: A custom-made section that reduces the diameter of the chamber from 12 inches to 6 inches. It is made from steel. The edges are welded to provide a seal. Lastly, (4) the aerosol sampling section: This is a custom fabricated, 6-inch-diameter, 2-foot-long duct-shaped section that is a single piece of steel. Six inches from the edge it contains a sampling port, where a sampling port/device was connected.

All sections of the experimental setup were connected through gasket fittings. Certain sections were sealed with silicone HVAC sealant and HVAC seal tape.

### 2.3. Measurement of Effective Airflow Inside Chamber

The Fan/filtration Unit produces a maximum flowrate of 550 cfm, as per the manufacturers’ ratings. To determine the actual flowrate inside the experimental chamber, an anemometer (TSI Inc., Shoreview, MN, USA) was utilized. This anemometer contains a probe that was placed inside the duct through the injection and sampling ports. The probe was placed at the center of the chamber duct and the flowrate was measured at the injection and sampling ports. Six distinct airflow readings were obtained and averaged to obtain the airflow.

### 2.4. Measurement of Ion Concentration Inside Chamber

To measure the concentration of ions inside the experimental chamber, an ionometer (AlphaLab AIC2, Salt Lake City, UT, USA) was utilized. The ionometer was placed inside the chamber, in the upper part of the ‘U’-shaped section, at 6 inches from the ionizer (see Figure 1). The entire experimental duct was connected to ground, in order to avoid any electrical interference. The ion measurement was performed for two conditions: (1) without any aerosol being introduced into the experimental system, and (2) with aerosol being introduced into the system. The second condition was utilized to assess the concentration of ions generated in the presence of bioaerosol and accommodate of any potential ion losses due to any potential changes in relative humidity.

A Collison nebulizer (CH Technologies, Westwood, NJ, USA) was connected to the inlet of the duct for introducing the aerosol into the system. A solution of 1% NaCl was added to the nebulizer to produce aerosol particles that would mimic viral particles. The ionizer was turned on and measurements were performed in a continuous manner for 5 min. Temperature and relative humidity (RH) inside the system were monitored during the measurement. Ozone levels were also measured with an Ozone monitor (2B Technologies, Broomfield, CO, USA).

### 2.5. Viral Strain Utilized for Efficacy Testing

Bacteriophage MS2 was utilized as a representative of non-enveloped viruses [26,27,28,29,31,32]. It has been utilized as a surrogate virus for assessing the antiviral efficacy of various technologies [17,26,28,33,34,35]. It is one of the oldest models in modern molecular microbiology, with the virus having been fully characterized [36]. Despite the fact that MS2 is an intrinsic non-enveloped virus of the Leviviridae family, it has been shown that it could be used as safe surrogate for enveloped viruses and quasi-enveloped viruses as well. A review of possible approaches towards SARS-CoV-2 studies states that, when taking into account the morphological and genetic properties, the phages of the Leviviridae family can be considered to be the best surrogates for coronavirus [37]. The bacteriophage MS2 was acquired from ATCC (Strain no. 15597-B1, American type culture collection, Manassas, VA, USA). Bacteriophage MS2 stock of concentration 10^10^ pfu/mL was stored in tryptic soy broth (TSB; Hardy Diagnostic, Santa Maria, CA, USA) supplemented with 0.1% glucose, 2 mM CaCl_2_, and 10 μg/mL thiamine at −80 °C.

### 2.6. Generation of Viral Bioaerosol

A single-jet Collison nebulizer (CH Technologies, Westwood, NJ, USA) containing 1 × 10^10^ pfu/mL of bacteriophage MS2 in Phosphate-Buffered Saline (PBS) (VWR, Radnor, PA, USA) was connected to the input port of the duct (as described earlier). The nebulizer was operated at 40 psig (pound-force per square inch) input pressure. At this input pressure, the nebulizer produced viral aerosol at a flowrate of 3.3 lpm. The injection of viral aerosol was perpendicular to the flow of air inside the duct.

### 2.7. Sampling of Viral Bioaerosol

An SKC Biosampler (SKC Inc., Eighty-Four, PA, USA) was utilized for the sampling of the viral aerosol from the experimental system as shown in Figure 1. Five ml of PBS was added to the collection jar. A vacuum pump was connected to the biosampler to provide the negative flowrate required for sampling. The SKC biosampler was connected to the sampling port, and it was operated at a flowrate of 12.5 lpm.

### 2.8. Experimental Protocol for Ionizer Viral Inactivation Tests

The fan/filter (VEVOR) system was operated at the highest airflow rate. The nebulizer was operated at 40 psi of air pressure. The viral aerosol flow was allowed to stabilize for a 5 min interval. At 5 min after the initiation of the viral aerosol injection, the biosampler was tuned on, and sampling was performed continuously for 5 min. After 5 min of sampling, the sampled solution was removed from the biosampler collection cup and added to a centrifuge tube for further analysis. This was considered a control sample. Fresh sampling fluid was added to the collection cup of the sampler. The sampling was repeated two more times with an identical protocol. In total, three control samples were obtained.

The viral aerosol injection continued with the nebulizer operation. After a predetermined time interval, the ionizer device was turned on. The device was operated for 5 min pre-sampling. At 5 min after ionizer initiation, the biosampler was tuned on and sampling was performed continuously for 5 min. After 5 min of sampling, the sampled solution was removed from the biosampler collection cup and added to a centrifuge tube for further analysis. This was considered as an ionizer test sample. Fresh sampling fluid was added to the collection cup of the sampler. The sampling was repeated two more times with the exact same protocol. In toto, three test samples were obtained. Temperature and relative humidity were monitored throughout the experiments.

### 2.9. Plaque Assay for Bacteriophage MS2 Quantification

*Escherichia coli* C-3000 strain (Strain no. 15597, American Type Culture Collection, Manassas, VA, USA) was utilized for the quantification of the bacteriophage MS2. An overnight culture of *E. coli* C-3000 was diluted 1:100 in tryptic soy broth (TSB) supplemented with 0.1% glucose and 10 μg/mL thiamine. It was then incubated at 37 °C with shaking until OD_600_ reached 0.4–0.6 (log phase growth). Agar plates with 1% Tryptic Soy Agar (TSA) were prepared beforehand. During the assay, a 0.5% TSA solution was prepared as the overlay agar. A total of 9 mL of this 0.5% TSA overlay agar was aliquoted and held at 50 °C. Serial dilutions of the control and ionizer test samples were carried out. To each 9 mL tube of 0.5% TSA, 0.3 mL of the log phase *E. coli* C-3000 culture was added, as well as 36 uL of supplement B (0.1% glucose, 2 mM CaCl_2_, and 10 μg/mL thiamine) and 0.7 mL of each dilution of test samples. The mixture was vortexed and poured onto 1% TSA-bottom agar plates. The plates were incubated at 37 °C overnight. Plaques were counted, and the pfu/liter of air was calculated, utilizing the dilution factor of the sample and the total air volume sampled. The lowest limit of detection for this assay was calculated as 0.3428 pfu/liter of air.

### 2.10. Statistical Analysis

For airflow and ion measurements, six individual measurements were conducted. The average and standard deviation of each were considered as data points. Each inactivation experiment was repeated in triplicate. The arithmetic means of triplicates were utilized as data points. The standard deviation was used as the error.

## 3. Results and Discussion:

### 3.1. Measurement of Effective Airflow Inside Duct

The effective airflow inside the duct was measured as mentioned in the methods section. The airflow at the sampling port was measured as 298.45 (±3.38) cfm. For the calculation of residence time of bioaerosol in contact with the ions, this flowrate was considered. This airflow of 298.45 (±3.38) cfm falls well within the operating range of both of the ionizer devices, as stated in Section 2.1 above.

The residence time of the bioaerosol in contact with the ions was calculated as 0.625 s. This was calculated according to airflow in each section of the duct between the ionizer section where the ions were generated and the bioaerosol sampling port.

### 3.2. Measurement of Ion Concentration Inside Duct

The concentration of ions produced by the ionizers inside the experimental duct was measured with an ionometer. For each individual ionizer, two sets of ion level measurements were conducted. The results are presented in Table 1. For ionizer device A, the concentration of ions without any presence of bioaerosol was 2.836 (±0.16) × 10^5^/cc. This was reduced to 2.546 (±0.31) × 10^5^/cc in the presence of bioaerosol. This represents a 10% ion loss in the presence of bioaerosol, albeit not statistically significant (*p* = 0.2233). The temperature and humidity during the experiments was 21 °C and 42%, respectively. No significant change in either temperature or RH was observed during ion measurements. For device B, without the presence of any bioaerosol, the concentration of ions produced was 1.578 (±0.15) × 10^6^/cc. This reduced to 1.116 (±0.05) × 10^6^/cc in the presence of bioaerosol produced by the nebulizer and introduced into the system. Here, the reduction in levels of ions was higher and statistically significant, as compared to device A, with a 29.2% reduction in presence of the aerosol (*p* = 0.0072). The temperature and humidity during the experimental rating of the ionizer B were 20 °C and 45%, respectively. For both ionizers, the temperature varied by <±1 °C and the relative humidity varied by <±1.5%. This variation was not considered statistically significant. The reductions in the concentrations of ions can be attributed to collisions with aerosol particles. Ozone was below the detection limit of the measurement device (3 ppb).

### 3.3. Bacteriophage MS2 Inactivation

**Figure 2:**
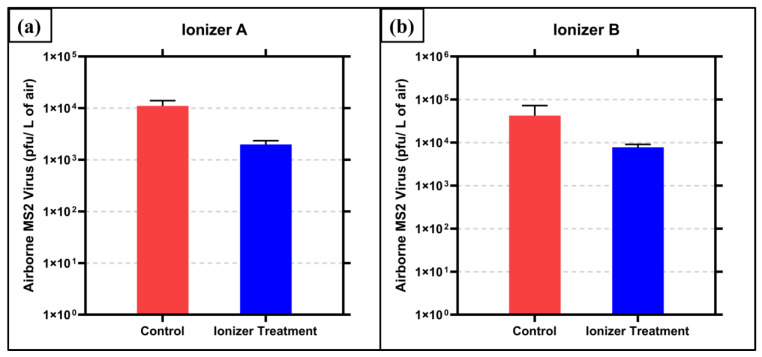
Inactivation of airborne MS2 by ions generated by air ionizer (**a**) device A and (**b**) device B. Averages of triplicate runs are graphed with ± 1 s.d. as error.

The results of the bacteriophage MS2 inactivation testing with Ionizer A exposure are shown in Figure 2a. The control (no ionizer exposure) air samples contained 1.1 × 10^4^ (±2.46 × 10^3^) pfu/liter of air of bacteriophage MS2. The air samples exposed to ions generated by ionizer A contained 1.98 × 10^3^ (±3 × 10^2^) pfu/liter of air. This translates to an 82.02% (0.745 log_10_) reduction (*p* = 0.0032). The temperature and relative humidity were measured to be 21 °C and 56%, respectively.

The results of the bacteriophage MS2 testing with Ionizer B exposure are shown in Figure 2b. The control (no ionizer exposure) air samples contained 4.23 × 10^4^ (±2.48 × 10^4^) pfu/liter of air of bacteriophage MS2. The air samples exposed to ions generated by ionizer B contained 7.73 × 10^3^ (±1.09 × 10^3^) pfu/liter of air. This translates to an 81.72% (0.738 log_10_) reduction (*p* = 0.0743). The temperature and relative humidity were measured to be 20 °C and 69%, respectively.

The results from the bacteriophage MS2 inactivation indicate significant inactivation for ionizer A, with *p* < 0.05. For ionizer B, the results are significant only to the 0.05 < *p* < 0.1 level; however, the scientifically meaningful nature of results can be attributed to the fact that contact time for the ions and the airborne virus is 0.625 s. This residence time was calculated according to the effective airflow in each section of the duct, between the ionizer section where the ions were generated and the bioaerosol sampling port, where the bioaerosol exited the duct. Since this is a single pass system and the effective flow rate was high (298 cfm), the residence time was quite short. According to ASHRAE standards, the required flow rate for indoor spaces is 15 cfm/person [38,39]. Hence, the flow rate utilized here corresponds well to the HVAC duct of a 20-person occupancy room, where one would expect such short residence times.

There have been few studies that have investigated the efficacy of technologies against airborne bacteriophage MS2. As the experimental parameters (such as the flow rate, residence time, and power consumption) vastly differ for studies on ionizers, direct comparison of the inactivation efficiencies is difficult. A study was conducted by Hyun et al., to determine the efficacy of corona discharge-generated bipolar ions. The experimental setup utilized by them included a test duct with a 0.04 × 0.04 m^2^ cross-sectional area and a length of 1 m. This was a design similar to that of the present study. The ionization plasma source was installed inside the duct, and bioaerosol of MS2 was exposed to it. The difference in methodology, however, was that in their study, much larger contact times, of 15 and 30 min, were utilized [17]. This was primarily due to the effective flow rate utilized by them being magnitudes lower than the one utilized in the present study. Wu et al. exposed airborne MS2 to atmospheric pressure cold plasma, which is known to generate ions, and for a short exposure time of 0.12 s, 80–95% inactivation of MS2 was reported, corresponding to 20–28 Watts of plasma power. Although this amount of inactivation is similar to the numbers seen in present study, the power utilized by their system is higher than that of the ionizers tested here [40]. Another study utilized nanosilver/TiO_2_ filters with a negative air ionizer and discovered that the highest efficacy could be found with a combination of two technologies, for a 97% removal of bacteriophage MS2 aerosol [35]. For ozone-based inactivation, a study reported that a dose of 3.43 ppm was required to inactivate 90% of MS2 bioaerosol, with 13.8 s contact time, thus emphasizing that ozone produces much slower inactivation as compared to the ions presented here [41]. In terms of UV as an intervention method, in an earlier study by the authors, a commercially available UV-based 222 nm device was found to produce a 90% reduction in the concentration of airborne HCoV-229E after 7.2 s of residence time [9].

When considering nanotechnology-based methods, the authors have developed a novel nano-carrier aerosol called Engineered Water Nanostructures (EWNS) for inactivation of viruses in air. In an earlier study, EWNS generated with nanogram levels of hydrogen peroxide produced a 94% reduction in the concentration of airborne H1N1/PR/8 Influenza virus [16]. The EWNS nano-carrier technology has also been shown to be effective against mycobacteria, as it produced a 68% reduction in the concentration of airborne *Mycobacterium parafortuitum* (a surrogate of *Mycobacterium tuberculosis*) after exposure to 36,000 particle of EWNS/cc [42]. Earlier mechanistic studies which evaluate ionizers and related ion generating cold plasma devices have pointed out ions, as well as reactive oxygen species (ROS) and ozone to be the major inactivating agents [11,43,44]. Here, in this study, ozone can be eliminated as the ozone generated was lower than the detection limit of the monitors utilized, i.e., lower than 3 ppb. This points to ions and ROS as potential inactivating agents. Further investigation into the exact mechanism is warranted.

It should also be considered here is that String et al., in their study of the various surrogates utilized for SARS-CoV-2 found that the bacteriophage MS2 is more difficult to inactivate, as compared to the SARS-CoV-2 [27]. This is not surprising, given that SARS-CoV-2 is an enveloped virus and MS2 is a small, non-enveloped virus, and it is generally accepted that enveloped viruses are more highly susceptible to chemical inactivation than small, non-enveloped viruses [45]. Thus, it is suggested that the efficacies of the ionizer devices tested in the present study are expected to be even higher when challenged with the SARS-CoV-2 virus.

## 4. Conclusions

The resent study demonstrates the efficacy of air ionizers as an intervention technology against a surrogate of SARS-CoV-2. The results indicate that such devices can indeed be installed as an engineering intervention for arresting the spread of viruses. Such experimental research is needed in the public health space, in order to develop and implement successful antiviral intervention technologies in the future.

## Figures and Tables

**Table 1:** Concentrations of ions inside the chamber as generated by ionizer devices A and B.

Experimental Conditions	Device A Ions (#/cc)	Device B Ions (#/cc)
No Aerosol Injection (Nebulizer OFF)	2.836 (±0.16) × 10^5^	1.578 (±0.15) × 10^6^
Aerosol Injection (Nebulizer ON)	2.546 (±0.31) × 10^5^	1.116 (±0.05) × 10^6^

## Data Availability

The original contributions presented in the study are included in the article; further inquiries can be directed to the corresponding author.
